# Thoracic radiation in combination with erlotinib—results from a phase 2 randomized trial

**DOI:** 10.3389/fonc.2024.1412716

**Published:** 2024-08-01

**Authors:** Hanne Marte Nymoen, Tine Norman Alver, Henrik Horndalsveen, Hanne Astrid Eide, Maria Moksnes Bjaanæs, Odd Terje Brustugun, Bjørn Henning Grønberg, Vilde Drageset Haakensen, Åslaug Helland

**Affiliations:** ^1^ Institute for Cancer Research, Department of Cancer Genetics, Oslo University Hospital, Oslo, Norway; ^2^ Department of Clinical Medicine, University of Oslo, Oslo, Norway; ^3^ Department of Oncology, Oslo University Hospital, Oslo, Norway; ^4^ Section of Oncology, Drammen Hospital, Vestre Viken Health Trust, Drammen, Norway; ^5^ Department of Clinical and Molecular Medicine, Norwegian University of Science and Technology, Trondheim, Norway; ^6^ Department of Oncology, St Olavs Hospital, Trondheim University Hospital, Trondheim, Norway

**Keywords:** non-small cell lung cancer, radioresistance, EGFR-inhibitor, erlotinib, adverse events, toxicity, quality of life

## Abstract

**Background:**

Radiotherapy (RT) can be used to reduce symptoms and maintain open airways for patients with non-small cell lung cancer when systemic treatment is not sufficient. For some patients, tumor control is not achieved due to radioresistance. Concurrent inhibition of epidermal growth factor receptors has been proposed as a strategy to overcome radioresistance but may increase toxicity. We performed a randomized trial to assess the efficacy, tolerance, and quality of life of concurrent erlotinib and palliative thoracic RT for patients with advanced non-small cell lung cancer.

**Methods:**

Patients were randomized 1:1 to RT alone (arm A) or in combination with erlotinib (arm B). A computed tomography (CT) scan at baseline and one at 4–12 weeks after inclusion was used to evaluate treatment response. Adverse events were registered during treatment and the subsequent 30 days. Health-related quality-of-life questionnaires were completed by the patients at baseline, weeks 2, 6, and 20.

**Results:**

A total of 114 patients were included. Of the 74 patients with CT scans available for evaluation of treatment effect, there were no significant differences in tumor size reduction between the two groups: median 14.5% reduction in the control arm A and 17.0% in the erlotinib arm B (*p* = 0.68). Overall survival was not significantly different between the two treatment arms: 7.0 and 7.8 months in arm A and arm B, respectively (log-rank *p* = 0.32). There was no significant increase in adverse events in the experimental arm, other than what is expected from erlotinib treatment alone. Overall, patients reported similar quality of life in both treatment arms.

**Conclusion:**

Concurrent erlotinib and palliative thoracic RT for patients with advanced non-small cell lung cancer was well tolerated but did not improve the efficacy of the RT.

**Clinical trial registration:**

ClinicalTrials.gov, identifier NCT02714530.

## Introduction

More than 2.2 million people are diagnosed with lung cancer worldwide each year, and almost 1.8 million die from the disease, making it the largest contributor to cancer deaths (1). A large proportion (39%) is diagnosed with advanced disease, for whom 5-year survival is only 6.1% (2). Better treatment options for these patients are needed.

Radiotherapy (RT) is used for patients with advanced non-small cell lung cancer (NSCLC) for symptom relief or to prevent tumors from affecting vital organ function, commonly obstruction of central airways. A phase 3 study of 421 advanced lung cancer patients treated with palliative RT to the thorax reported symptom improvement in 40%–50% of patients with respect to dyspnea and cough, and 80%–90% of patients with respect to hemoptysis (3). However, for some patients, tumor control is not achieved due to radioresistance.

The epidermal growth factor receptor (EGFR) is a transmembrane glycoprotein with a tyrosine kinase belonging to the ErbB family (4). It is primarily found on cells of epithelial origin and is often overexpressed in cancer cells (5). Activation of EGFR signaling leads to increased cell growth, proliferation, invasion, angiogenesis, and metastasis (6). EGFR inhibitors exist both as monoclonal antibodies and as tyrosine kinase inhibitors (TKIs). TKIs bind to the intracellular part of the receptor to block the phosphorylation, hence blocking the activation of EGFR signaling. Erlotinib is a TKI used in the treatment of several cancer types, including NSCLC, and was first approved by the U.S. Food and Drug Administration in 2004 (7).

Studies have shown that cancer cells can upregulate EGFR signaling when exposed to irradiation (8). Inhibiting the EGFR pathway through EGFR inhibitors has been proposed as a possible strategy to reduce radioresistance, and preclinical models have shown enhanced radiosensitivity when combining RT with EGFR inhibition. The combination appears to increase the rate of apoptosis and induce cell cycle arrest in the G2/M phase (9–12). Furthermore, a phase 3 trial of advanced head and neck cancer showed that concurrent cetuximab prolonged survival compared with RT alone (13, 14).

Based on these data, we designed a trial to investigate whether palliative RT combined with erlotinib increased local tumor response in advanced NSCLC, compared to RT alone. Erlotinib was selected since it was one of two EGFR inhibitors approved for patients with NSCLC regardless of EGFR mutations in Norway (the other was gefitinib) at the time when the study was designed (15). The other aims were to investigate whether the combination prolonged overall survival (OS), increased toxicity, and the effect on quality of life (QoL). The primary endpoint was to determine if erlotinib given orally along with concurrent external beam radiation therapy, prolonged local tumor control compared to treatment with external beam radiation therapy alone. Secondary endpoints included evaluation of safety of the combination of erlotinib and RT, health-related QoL measurements, and OS.

## Materials and methods

### Study population and study design

Patients with an advanced, histologically confirmed NSCLC, referred for palliative RT to the hilus/mediastinum and with an Eastern Cooperative Oncology Group (ECOG)—status of 0–2 were eligible for the study (all eligibility criteria are listed in **Supplementary File S1**, **Supplementary Table S1**). The randomization was 1:1 between external beam fractionated RT alone (arm A) or concurrent erlotinib and RT (arm B). Randomization was done in blocks of 7, stratified on ECOG status.

### Treatment

RT was given to the mediastinum/hili by two lateral opposing six MV photon beams of 30 Gy in 10 fractions, 5 days a week. This was the standard palliative fractionation regimen at the time.

Patients in arm B were given erlotinib 150 mg QD orally from the day before radiation until the last day of radiation unless intolerable side effects or patients wanted to discontinue.

### Clinical outcomes

Computed tomography (CT) scans at baseline and follow-up (ranging from 4 to 12 weeks after inclusion) were used for response assessment. Local tumor response was evaluated by measuring the largest tumor diameter of the central mediastinal tumor volume (lymph node conglomerates included) at the same anatomical position in the baseline CT and follow-up scans for each patient. The percentage change was calculated. Toxicity was evaluated by recording of adverse events during treatment and for the subsequent 30 days and graded according to the National Cancer Institute Common Terminology Criteria for Adverse Events (CTCAE) version 4.0. Patients completed the European Organization for Research and Treatment of Cancer (EORTC) Quality of Life Group Core Questionnaire (EORTC QLQ-C30), version 3.0, and the lung cancer–specific questionnaire EORTC QLQ-LC13 at baseline, weeks 2, 6, and 20 after inclusion.

### Statistical analysis

The trial was designed to detect a 2-month (50%) or greater improvement due to the addition of erlotinib. The statistical significance and power were set at 5% and 80%, respectively, for a one-sided test, to detect an approximate 2-month increase in median survival with the addition of erlotinib, on an intention to treat basis. Seventy-five patients were to be assigned to each treatment arm, totaling 150 patients in the study. Two hospitals in Norway (Oslo University Hospital and St. Olavs Hospital) recruited patients to the trial, starting in 2012.

Due to the lack of normal distribution in arm B, a Wilcoxon signed-rank test was performed to estimate the percentage differences in the largest tumor diameter between arm A and arm B and between groups of EGFR mutation negative and EGFR mutation unknown status on an intention-to-treat basis. OS was calculated from the day of randomization until death from any cause. A log-rank test was performed to compare the survival difference between the groups. Cox regression was used to estimate the hazard ratio. A two-sided significance level of 0.05 was used. The study was powered to detect a 50% longer disease control in the radiation field for patients treated with concomitant erlotinib. The raw scores from the two EORTC questionnaires were grouped and transformed to a 0–100 scale, as recommended by the EORTC scoring manual (16). In accordance with King et al. and Osoba et al, a 10-point difference between the scores in time or between the two study arms was seen as a clinically relevant difference (17, 18). The statistical analyses were done using RStudio, version 1.4.1717.

### Ethical approval

Informed consent was obtained from all participants. The study was approved by the Regional Ethics Committee in South-East of Norway (reference number 2012/320), the Norwegian Medicines Agency (Eudra-CT no 2012-000967-25), and the Radium Hospital internal review board. The trial is registered in ClinicalTrials.gov with ID: NCT02714530.

## Results

### Patient characteristics

A total of 114 patients were included in the study from Oslo University Hospital and St. Olavs Hospital, Trondheim University Hospital, in the period 2012–2019, 57 patients in arm A (RT alone) and 57 patients in arm B (RT plus erlotinib). The inclusion was terminated prior to reaching the planned 150 patients due to a change in routine fractionation of palliative RT reducing the number of eligible patients considerably. Patient characteristics are shown in **Table 1**. The two arms were balanced with regards to sex, age, prior treatment, ECOG performance status, and disease stage. Based on local routine histology reports, there was a slightly higher percentage of patients with adenocarcinoma in arm B compared to arm A. Information about routine testing of EGFR mutation was available for 59 of the 71 adenocarcinomas, with no detected EGFR mutation.

**Table 1:** Characteristics, survival, and response for all patients and patients in each treatment arm.

Characteristics	Arm A, n = 57	Arm B, n = 57	Total, n = 114
Median age at inclusion, years (range)	70.3 (47.7–85.0)	69.2 (55.3–79.7)	69.4 (47.7–85.9)
Sex, number (%)			
Male	41 (72.9)	40 (70.2)	81 (71.1)
Female	16 (28.1)	17 (29.8)	33 (28.9)
ECOG performance status *n* (%)			
0	7 (12.3)	10 (17.5)	17 (14.9)
1	28 (49.1)	30 (52.6)	58 (50.9)
2	20 (35.1)	17 (29.8)	37 (32.5)
Missing	2 (3.5)	0 (0)	2 (1.8)
Stage *n* (%)			
2	1 (1.8)	1 (1.8)	2 (1.8)
3	9 (15.8)	7 (12.3)	16 (14.0)
4	44 (77.2)	49 (86,0)	93 (81.6)
Missing	3 (5.3)	0 (0)	3 (2.6)
Histology (%)			
Adenocarcinoma	30 (52.6)	41 (71.9)	71 (62.3)
Squamous	20 (35.1)	13 (22.8)	33 (28.9)
Other	7 (12.3)	3 (5.3)	10 (8.8)
Prior treatment			
Yes/No	37/ 20	34/ 23	71/ 43
Chemotherapy	19	18	37
Targeted	0	1	1
Immunotherapy	4	1	5
Thoracic RT	2	0	2
Thoracic surgery	3	7	10
RT/surgery brain	1	4	5
Median overall survival, months (range)	7.0 (0.1–82.0)	7.8 (0.9–89.4)	7.4 (0.1–89.4)
Male	7.0 (0.1–76.3)	6.7 (0.9–89.4)	6.8 (0.1–89.4)
Female	7.0 (0.4–82.0)	13.0 (1.7–76.6)	9.0 (0.4–82.0)
Tumour response Increase ≥ 20%	14 (19)	7 (12)	7 (12)
Decrease ≤ 30%	17 (23)	10 (17.5)	7 (12)

### Radiologic evaluation

Radiological follow-up was performed according to local routine. A total of 74 patients had both a baseline CT scan and an evaluation CT (ranging from week 4 to week 12), 34 patients in arm A and 40 patients in arm B, respectively.

### Local tumor control

**Figure 1:**
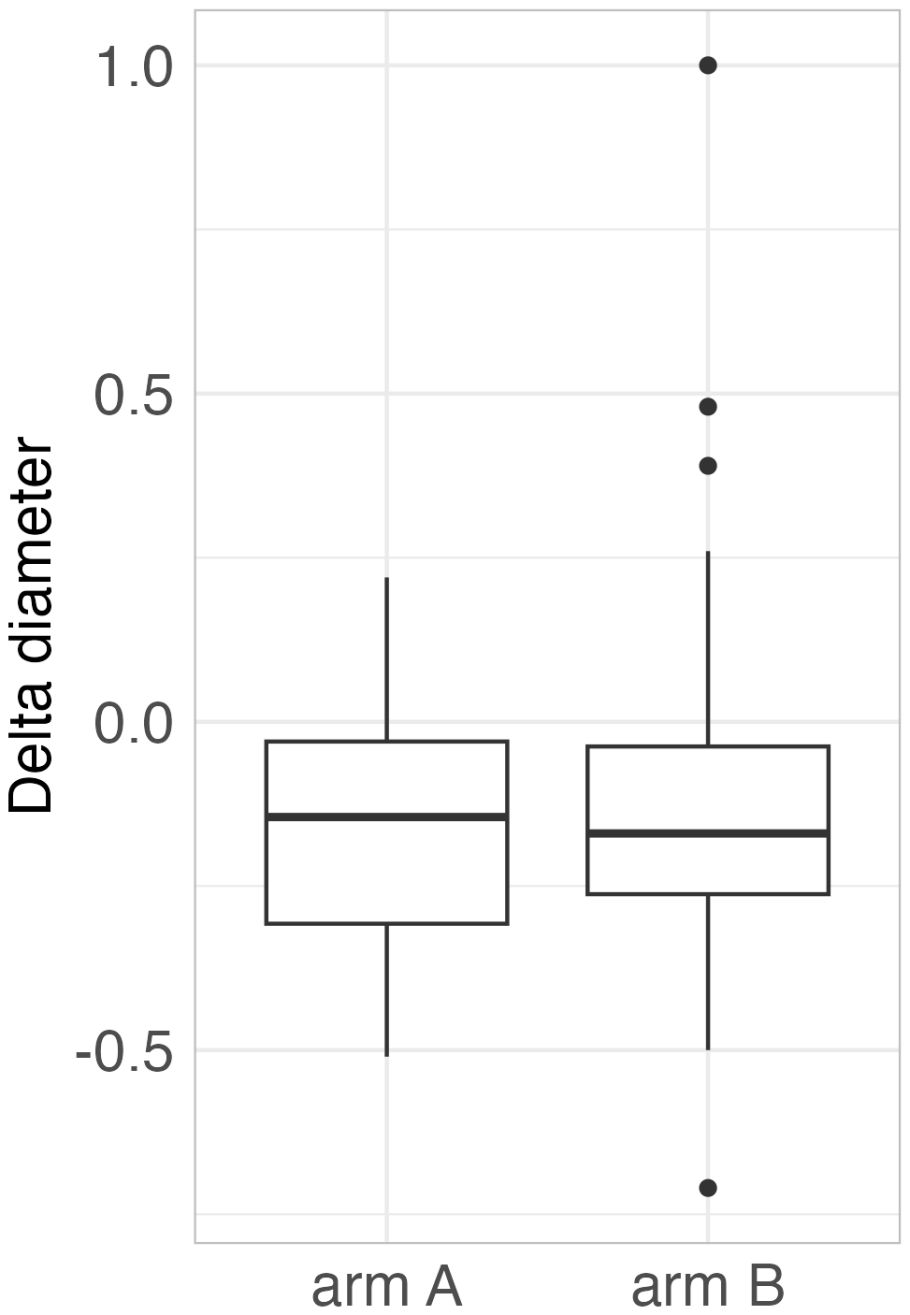
Change in tumor diameter from baseline to the first post-treatment CT in arm A (RT only) and arm B (erlotinib and RT).

The median change in tumor diameter (combined volume of mediastinal tumor mass) from baseline to the first post-treatment CT in all patients was −15.5% (standard arm A: −14.5%, erlotinib arm B: −17.0%, *p* = 0.68) (**Figure 1**). Similarly, there was no difference between the two arms in median change in tumor diameter for adenocarcinomas (*p* = 0.53). For the primary end-point, there were three patients with unknown EGFR status in the experimental arm B and five patients in the standard arm A. There was a trend towards longer response in the experimental arm than in controls, but the numbers are too small for a conclusive comparison (arm A: −13%, arm B: −24%, *p* = 0.46). There was no statistical difference in local control between patients in the erlotinib arm B with negative EGFR status (−13.0%, 28 patients) and patients with unknown EGFR mutation status (−24.0%, 3 patients), *p* = 0.18).

### Survival

**Figure 2:**
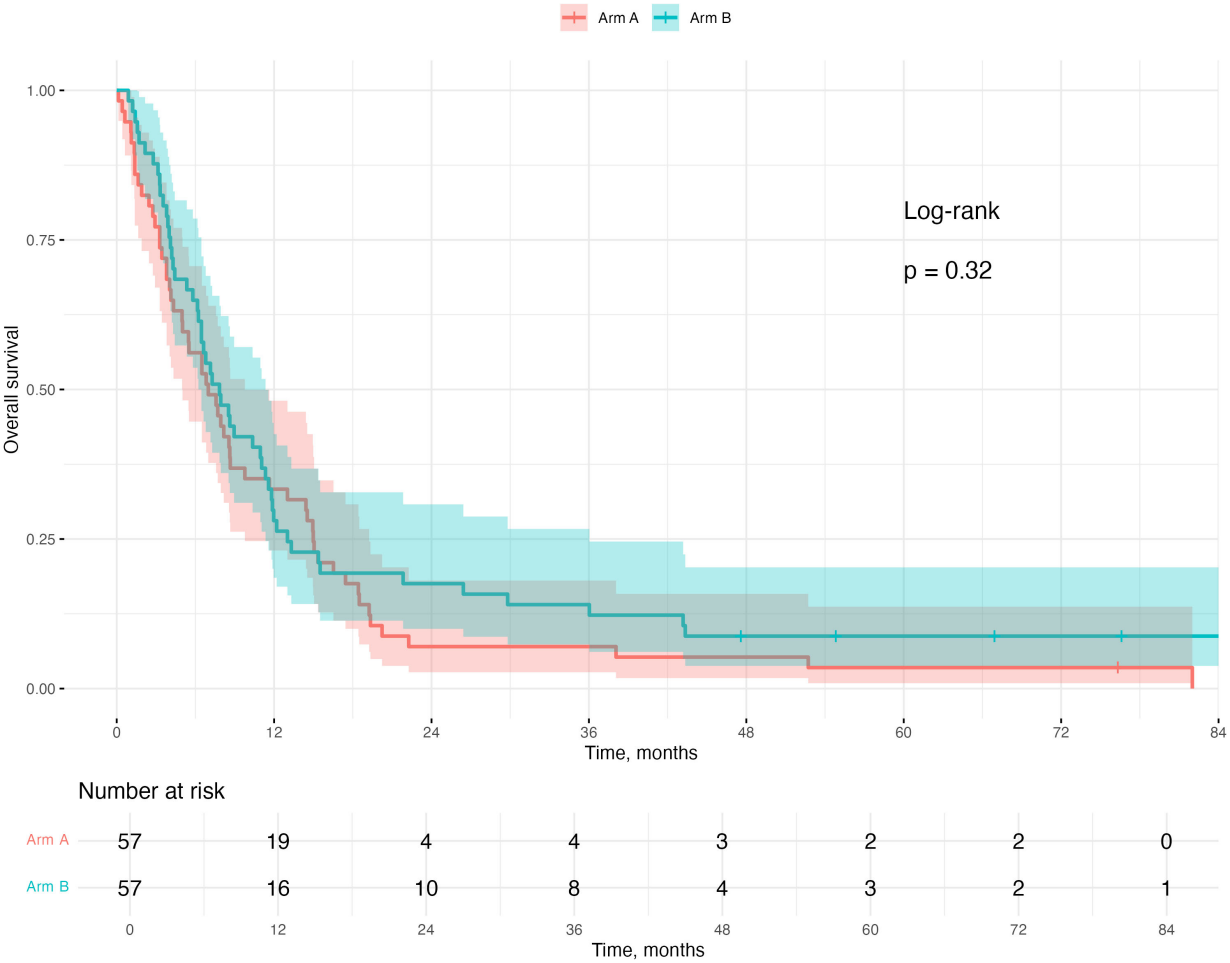
Kaplan–Maier plot of overall survival arm A (RT only) versus arm B (Erlotinib and RT).

At the censoring date, six patients were still alive, one in arm A and five in arm B. The median OS for the whole study cohort was 7.43 months [95% CI (6.46, 8.95)], 6.98 months [95% CI (4.98, 9.77)] in the standard arm A and 7.84 months [95% CI (6.23, 11.34)] in the erlotinib arm B (log-rank *p* = 0.32) (**Figure 2**).

Exploratory subgroup analyses confirmed established prognostic factors such as female gender and poor performance status and indicated a significantly shorter OS for the histological subtype “others,” compared to adenocarcinomas and squamous cell carcinomas (see **Supplementary File S1**, **Supplementary Figure S1**, and **Supplementary Table S2**).

### Adverse events

Adverse events of any cause and regardless of attribution to study treatment by the investigator were recorded for 112 of 114 patients and occurred in 40 (70.2%) of the patients in the standard arm A and in 51 (89.5%) of the patients in the erlotinib arm B. Grade 3 or higher adverse events occurred in 24.5% in standard arm A and 17.5% in erlotinib arm B. One patient in each arm died due to adverse events, both classified as unrelated to the study treatment (one case of atrial fibrillation and heart failure in the standard arm A and one case of thrombosis in the erlotinib arm B). Rash (57.9% vs. 0%), diarrhea (22.8% vs. 1.8%), and nausea/vomiting (19.3% vs. 10.5%) were reported more often in the erlotinib arm B, whereas dyspnea (14.0% vs. 12.2%), cough (10.5% vs. 7.0%), and thoracic pain (10.5% vs. 5.2%) occurred more often in the control arm. Adverse events leading to discontinuation of erlotinib occurred in 11 (19.3%) patients in the experimental treatment arm B, mainly (*n* = 7) due to acneiform rash grade 2 or 3. Specific adverse events reported in ≥5% of the study population are listed in **Table 2**.

**Table 2:** Adverse events.

	Arm A: RT only (n = 57)		Arm B: RT + erlotinib (n = 57)	
Event				
Any grade AE	40 (70.2%)		51 (89.5%)	
Grades 3–4 AE	13 (22.8%)		9 (15.8%)	
AE leading to treatment discontinuation	1 (1.8%)		11 (19.3%	
Specific AEs	Any grade	Grades 3–4	Any grade	Grades 3–4
Rash	0	0	33 (57.9%)	4 (7.0%)
Nausea/vomiting	6 (10.5%)	0	11 (19.3%)	0
Fatigue	11 (19.3%)	0	5 (8.8%)	0
Dyspnoea	8 (14.0%	2 (3.5%)	7 (12.2%)	1 (1.8%)
Esophagitis	8 (14.0%	0	7 (12.2%)	0
Diarrhea	1 (1.8%)	0	13 (22.8%)	0
Pain, other	5 (8.8%)	2 (3.5%)	8 (14.0%)	0
Cough	6 (10.5%)	0	4 (7.0%)	0
Thoracic pain	6 (10.5%)	2 (3.5%)	3 (5.2%)	0
Anorexia	5 (8.8%)	0	3 (5.2%)	0
Constipation	3 (5.2%)	0	4 (7.0%)	1 (1.8%)
Abdominal pain	2 (3.5%)	1 (1.8%)	4 (7.0%)	1 (1.8%)
Dizziness	1 (1.8%)	0	5 (8.8%)	0

### Health-related quality of life

Of the 114 patients included, 103 patients completed HRQoL questionnaires at one or more time points. At baseline, weeks 6 and 20, the questionnaires were completed by 87 of 114 (76%), 63 of 114 (55%), and 29 of 114 (25%) patients, respectively.

**Figure 3:**
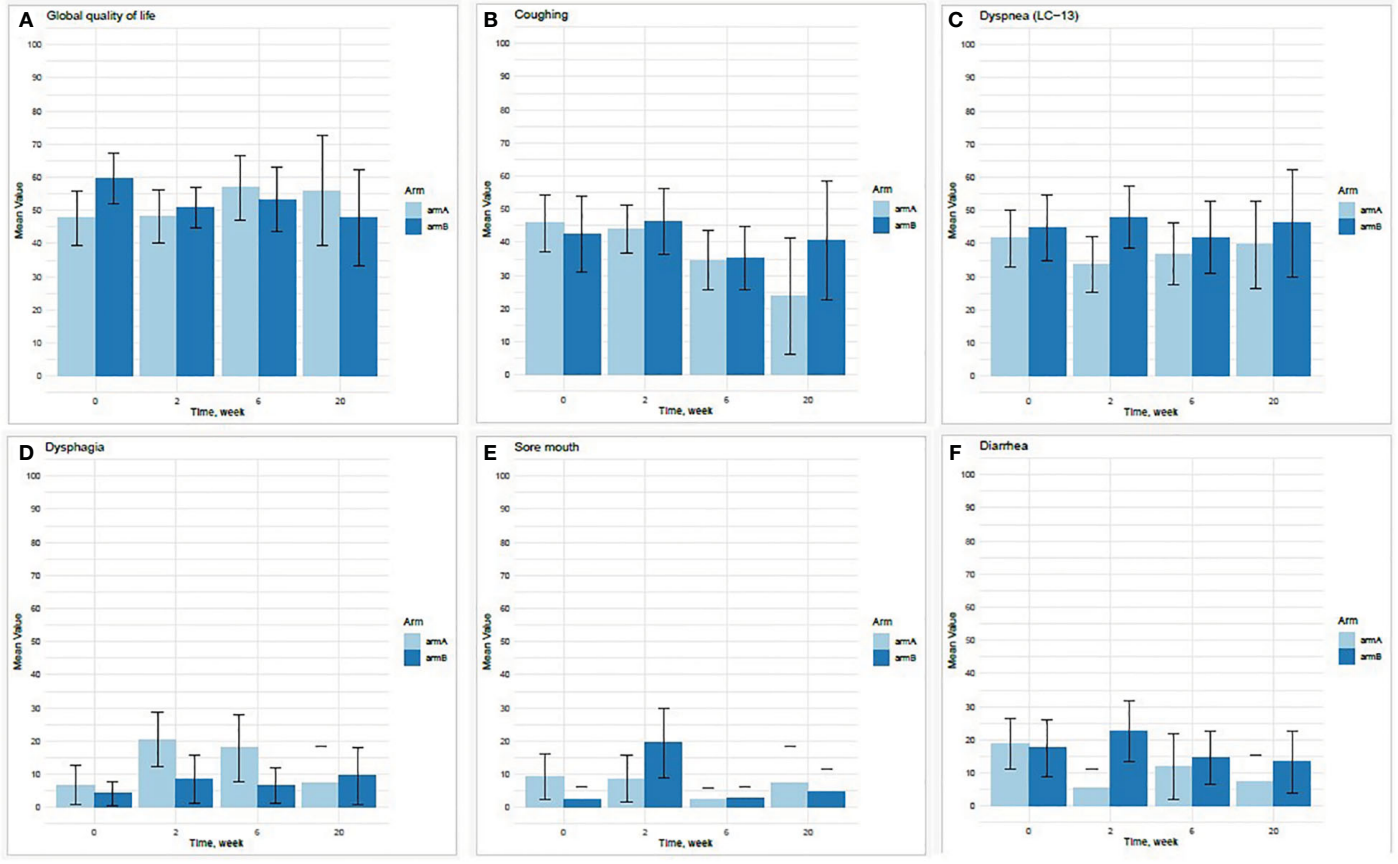
Mean scores at baseline, weeks 2, 6, and 20 for each treatment arm for selected variables from HRQoL questionnaires: global quality of life **(A)**, coughing **(B)**, dyspnoe (reported in two different questionnaires) **(C)**, dysphagia **(D)**, sore mouth **(E)**, and diarrhea **(F)**.

At baseline, there was a 12-point difference between the two study arms concerning global health status, with the erlotinib arm B (60 points) reporting better status than standard arm A (48 points) (**Figure 3A**). There was a trend towards reduced cough in both groups by week 6, while this trend was continued only in the control arm A (**Figure 3B**). Dyspnea was relatively stable during the course of the trial (**Figure 3C**). There was a trend towards increased dysphagia in weeks 2 and 6 for patients in arm A (**Figure 3D**) and increased sore mouth and diarrhea at week 2 for patients in the erlotinib arm B (**Figures 3E, F**). The 95% confidence intervals for all the HRQoL results were overlapping, reducing the robustness of the results (**Figure 3**; **Supplementary File S2**). All factors with a difference >10 points are listed in **Supplementary File S1** and **Supplementary Table S3**.

## Discussion

There was no significant difference in tumor response or OS between the group given RT alone versus the group given RT in combination with erlotinib. Further, there was no significant altered synergistic effect of the combinational treatment of erlotinib and RT together, with respect to adverse events or HRQoL.

During recent years, several clinical trials have combined EGFR inhibitors with RT to study if this could give a radio-sensitizing effect. Two of the trials added erlotinib to chemoradiation in stage 3 patients and reported promising results on toxicity and survival. A retrospective observational study of 60 pre-treated patients reported OS of 23 months (19) while a prospective study of 46 patients reported OS of 36 months, but did not meet the pre-study expectations (20). Despite this, both trials considered their results on OS promising and encouraged further trials. Two other single-arm studies concluded less favorably, reporting no increase in symptom relief or survival when adding erlotinib to palliative RT (21) and intolerable toxicity (22). A meta-analysis reported primary tumor control and OS in 16 single-arm studies where patients received EGFR TKIs concurrent with thoracic RT or chemoradiation therapy. They concluded that EGFR TKIs concomitant with thoracic RT or chemoradiation therapy might improve local tumor control and OS, but they also stressed that the evidence held low quality (23). The published trials are mostly single-arm studies with small sample sizes, limiting the interpretation of the clinical effect of a combined treatment. Further, differences in study design, inclusion criteria (including EGFR-mutation status), disease stage, and treatment regimens make comparison difficult (21, 24–27).

We have previously published data analyzing FDG-PET scans before, during, and after RT in 27 of the patients included in this trial (13 in arm A and 14 in arm B). We observed a higher metabolic activity in the tumors after RT in the combination arm B than in the control arm A, but our present results indicate that this does not translate into a significant difference in local control (28).

Patients with EGFR mutations are expected to respond to EGFR-TKI and inclusion of any patients with EGFR mutations would influence the results in this trial. Of 71 patients with adenocarcinoma in our study, EGFR mutation status was available for 59 patients, all without EGFR mutation. It is unlikely that patients with activating EGFR mutations were included in this study, based on information on the patients’ treatment received. Reflex testing of EGFR was recommended at the time of inclusion and patients with EGFR-positive tumors were offered TKIs outside this trial. Only one patient had earlier been treated with a TKI (afatinib) but stopped this treatment several months before inclusion in the study. We analyzed local control in patients with adenocarcinoma and unknown EGFR mutation status revealed slightly larger tumor shrinkage in patients treated with erlotinib, but the differences are not significant and the numbers are small. A larger shrinkage could indicate that some patients with unknown mutation status were EGFR positive.

We found erlotinib given concomitant with external beam RT to be safe during treatment and for the subsequent 30 days. There were no clear differences in AE and HRQoL other than that low-grade AEs related to erlotinib-treatment itself (rash and a trend toward more diarrhea/nausea/vomiting). Almost 20% of the patients in our study discontinued erlotinib due to adverse events; however, the threshold for stopping drug treatment was low. Grade 3, 4, or 5 AEs were not more common in the combined treatment arm. Since AEs were only registered until 30 days after completion of study treatment, our trial was not designed for capturing delayed toxicity including radiation pneumonitis.

These findings comply with results from other studies where erlotinib has been given in monotherapy or the combination of a TKI and RT has been tested (29–33) and indicate increased AEs of erlotinib with longer use (32).

In the two patient-reported EORTC questionnaires, C30 and LC-13, both treatment arms showed similar overall scores. While some scores differed by more than 10 points between study arms or time points, all confidence intervals were overlapping. This may be caused by limited power due to attrition during the course of the study and indicates that the results are not robust and cannot be used to draw final conclusions. The trends seen in reduced cough, deterioration in function, and temporary increase in sore mouth (the erlotinib arm B) and dysphagia (the control arm A) are as expected for this group of patients.

The main limitation of this study is the termination of the trial before 150 patients were included. This reduces the power of the study. The sample size estimation was optimistic from the start, the inclusion time was longer than anticipated, and recruitment was terminated. Despite not reaching the planned recruitment, the sample size is larger than what is seen in most of the other studies published. The patients are representative of the population of patients with NSCLC receiving palliative RT to the central airways at the time since RT is only given under the auspices of the public health care system in Norway.

Another limitation of the study is the lack of evaluable CT scans at a fixed point in time. There was no funding for additional CT scans in the trial, and scanning was performed according to local customs. Not all patients had a CT scan in the first three months after RT, reducing the number of patients available for the primary analyses even further. Limited treatment options for the patients implied a reduced clinical need for CT scans. The trial was not designed to collect further reports on AE 30 days subsequent to the treatment period and may not conclude about late-onset pneumonitis. After the study treatment, the patients were followed at their local hospitals for radiological controls. A clinical trial with larger sample size and more rigid radiological follow-up could provide data to conclude about the putative radiosensitizing role of erlotinib or other EGFR inhibitors. Given the weak data and the evolving field of radiosensitizers, we would not recommend another clinical trial to settle this.

## Conclusion

This randomized trial found no significant clinically relevant radiosensitizing effect of concomitant erlotinib and RT. Further, the trial showed that the combination did not give more systemic adverse events than what is expected with erlotinib treatment alone. Based on our data and the development in RT, we would not recommend further clinical trials on radiosensitizing effects of erlotinib, but rather search for other agents with the potential for greater effects.

## Data availability statement

The raw data supporting the conclusions of this article will be made available by the authors, without undue reservation.

## Ethics statement

The studies involving humans were approved by the Regional Ethics Committee in South-East of Norway (reference number 2012/320), The Norwegian Medicines Agency (EUDRACTNR. 2012-000967-25). The studies were conducted in accordance with the local legislation and institutional requirements. The participants provided their written informed consent to participate in this study.

## Author contributions

HN: Writing – original draft, Visualization, Formal analysis, Data curation, Supervision. TA: Writing – review & editing, Investigation, Formal analysis, Data curation. HH: Formal analysis, Writing – review & editing, Data curation. HE: Writing – review & editing, Investigation, Data curation. MB: Writing – review & editing, Investigation, Data curation. OB: Writing – review & editing, Investigation, Data curation. BG: Supervision, Writing – review & editing, Investigation, Data curation. VH: Supervision, Writing – review & editing, Investigation, Data curation. AH: Project administration, Writing – review & editing, Investigation, Funding acquisition, Data curation, Conceptualization.
