# Supplementary material for: Thoracic radiation in combination with erlotinib—results from a phase 2 randomized trial
Source: Front Oncol. 2024 Aug 1;14:1412716. doi: 10.3389/fonc.2024.1412716 (PMC11324589; doi:10.3389/fonc.2024.1412716)
Supplement: Supplementary file 1 [file DataSheet_1.docx]

**Supplementary File 1**

Table S1: Eligibility criteria

Figure S1: Overall survival female vs male

Table S2: Multivariate analysis, Cox regression Hazard Model

Table S3. Health-related Quality of Life

**Table S1: Eligibility criteria**

- Age > 18 years
- Histological or cytological verified NSCLC
- Palliative RT to the thorax indicated
- ECOG performance status 0-2
- Fertile patients must use contraception
- Signed informed consent
- Ability to understand and fill in QoL-questionnaires
- Capability to take per os medication
- Serum bilirubin < 2 times the upper limit of normal (ULN)
- AST and ALT < 2 times ULN (< 5 times ULN if liver metastases are present)
- Creatinine < 5 times ULN
- No pregnancy or nursing
- No other prior or concurrent malignant disease likely to interfere with the study treatment or comparisons
- No evidence of other significant laboratory findings or concurrent uncontrolled medical illness, that in the opinion of the investigator, would interfere with study treatment or results comparison or render the patient at high risk for treatment complications
- No prior RT to the same organ/place
- No concurrent treatment with other experimental drugs
- No known brain metastases in need of RT
- No known hypersensitivity to erlotinib or other substances in the erlotinib tablets

**Figure S1: Overall survival female vs male**


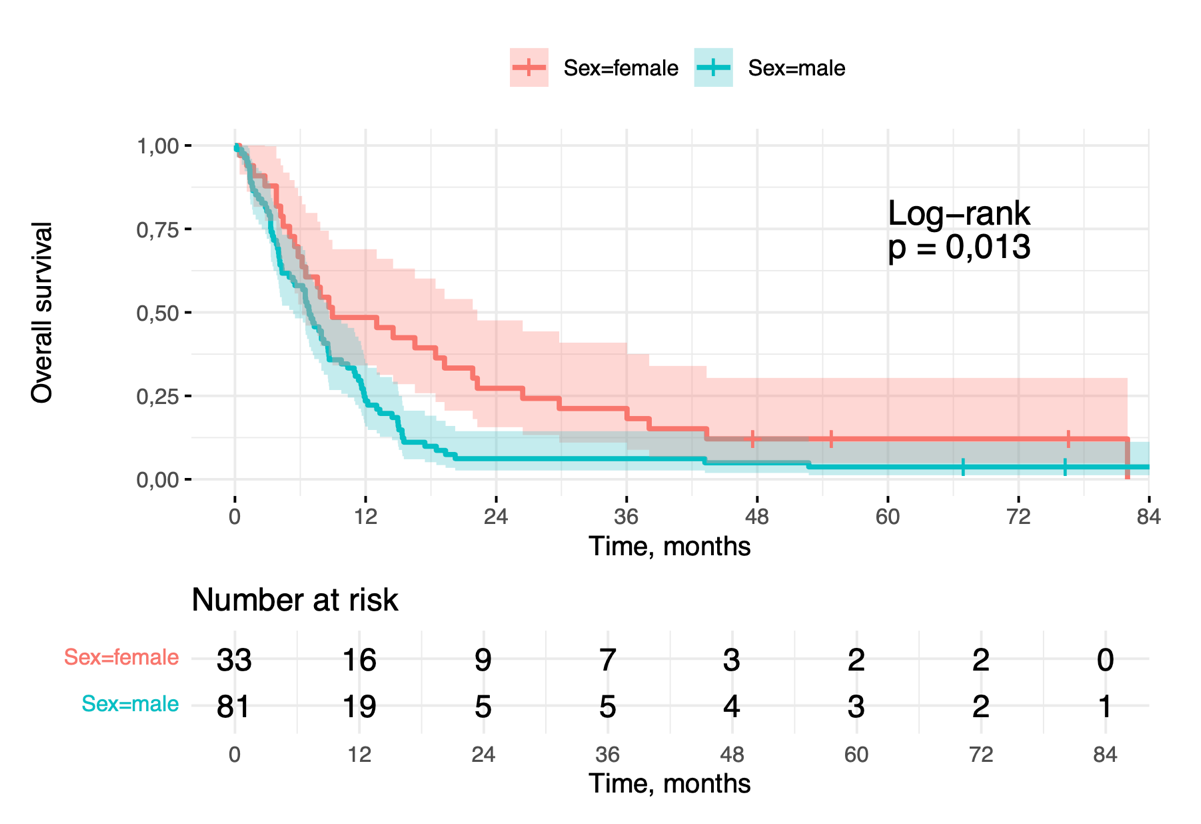


**Supplementary Table S2: Multivariate analysis, Cox regression Hazard Model**

|  | Hazard Ratio | Confidence interval | p-value |
| --- | --- | --- | --- |
| Arm |  |  |  |
| Arm A, RT Only | 1 |  |  |
| Arm B, Erlotinib + RT | 0.87 | 0.59 – 1.30 | 0.50 |
| Sex |  |  |  |
| Female | 1 |  |  |
| Male | 1.91 | 1.20 – 3.02 | 0.01 |
| Histology |  |  |  |
| Adeno carcinoma | 1 |  |  |
| Scquamous cell carcinoma | 0.94 | 0.60 – 1.47 | 0.78 |
| Other | 2.29 | 1.12 – 4.70 | 0.02 |
| ECOG |  |  |  |
| 0 | 1 |  |  |
| 1 | 1.71 | 0.91 – 3.23 | 0.10 |
| 2 | 2.24 | 1.16 – 4.35 | 0.02 |
| Stage |  |  |  |
| 2 | 1 |  |  |
| 3 | 0.57 | 0.13 – 2.59 | 0.47 |
| 4 | 0.82 | 0.20 – 3.40 | 0.78 |

**Supplementary Table S3.** **Health-related Quality of Life**

Selected scores from HR-QoL questionnaires that are different between treatment groups (with a difference >10 points). A higher score on the functioning scale (1) indicates better functioning, while a higher score on the symptom scale (2) reflects a higher symptom burden.

|  | Arm A, points | Arm B, points |
| --- | --- | --- |
| Baseline |  |  |
| Global health status (1) | 48 | 60 |
| Week 2 |  |  |
| Emotional function (1) | 70 | 82 |
| Pain (2) | 37 | 22 |
| Diarrhea (2) | 6 | 23 |
| Dyspnoea (LC-13) (2) | 34 | 48 |
| Sore mouth (2) | 9 | 19 |
| Dysphagia (2) | 20 | 8 |
| Week 6 |  |  |
| Insomnia (2) | 22 | 34 |
| Dysphagia (2) | 18 | 7 |
| Week 20 |  |  |
| Physical function (1) | 65 | 48 |
| Role function (1) | 62 | 32 |
| Dyspnea (C30) (2) | 52 | 62 |
| Cough (2) | 4 | 41 |
| Polyneuropathy (2) | 10 | 36 |
| Alopecia (2) | 2 | 14 |
