# Supplementary material for: Thoracic radiation in combination with erlotinib—results from a phase 2 randomized trial
Source: Front Oncol. 2024 Aug 1;14:1412716. doi: 10.3389/fonc.2024.1412716 (PMC11324589; doi:10.3389/fonc.2024.1412716)
Supplement: Supplementary file 2 [file DataSheet_2.pdf]

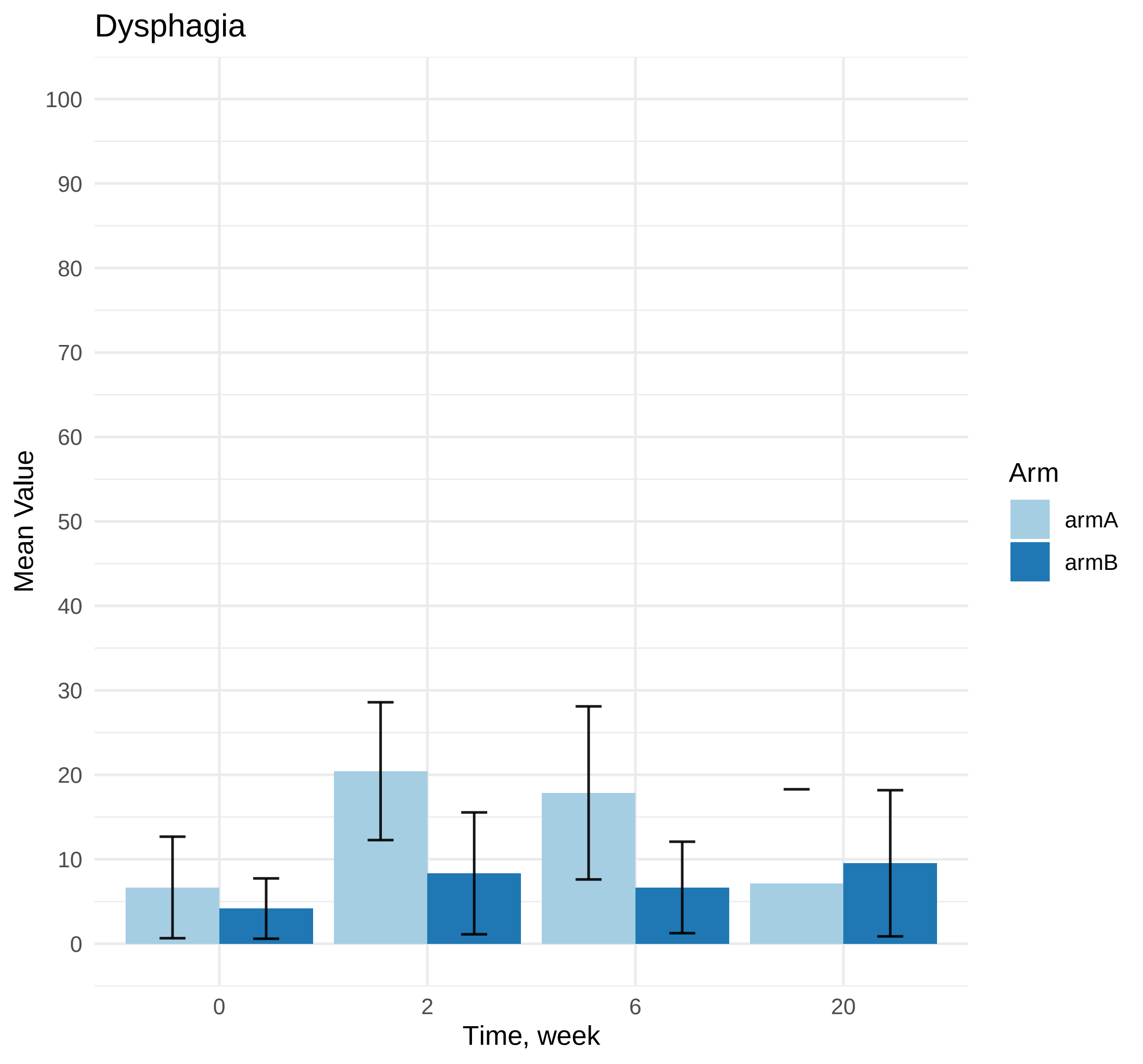
Dysphagia
Mean Value
100
90
80
70
60
50
40
30
20
10
0
0
2
6
20
Time, week
Arm
armA
armB

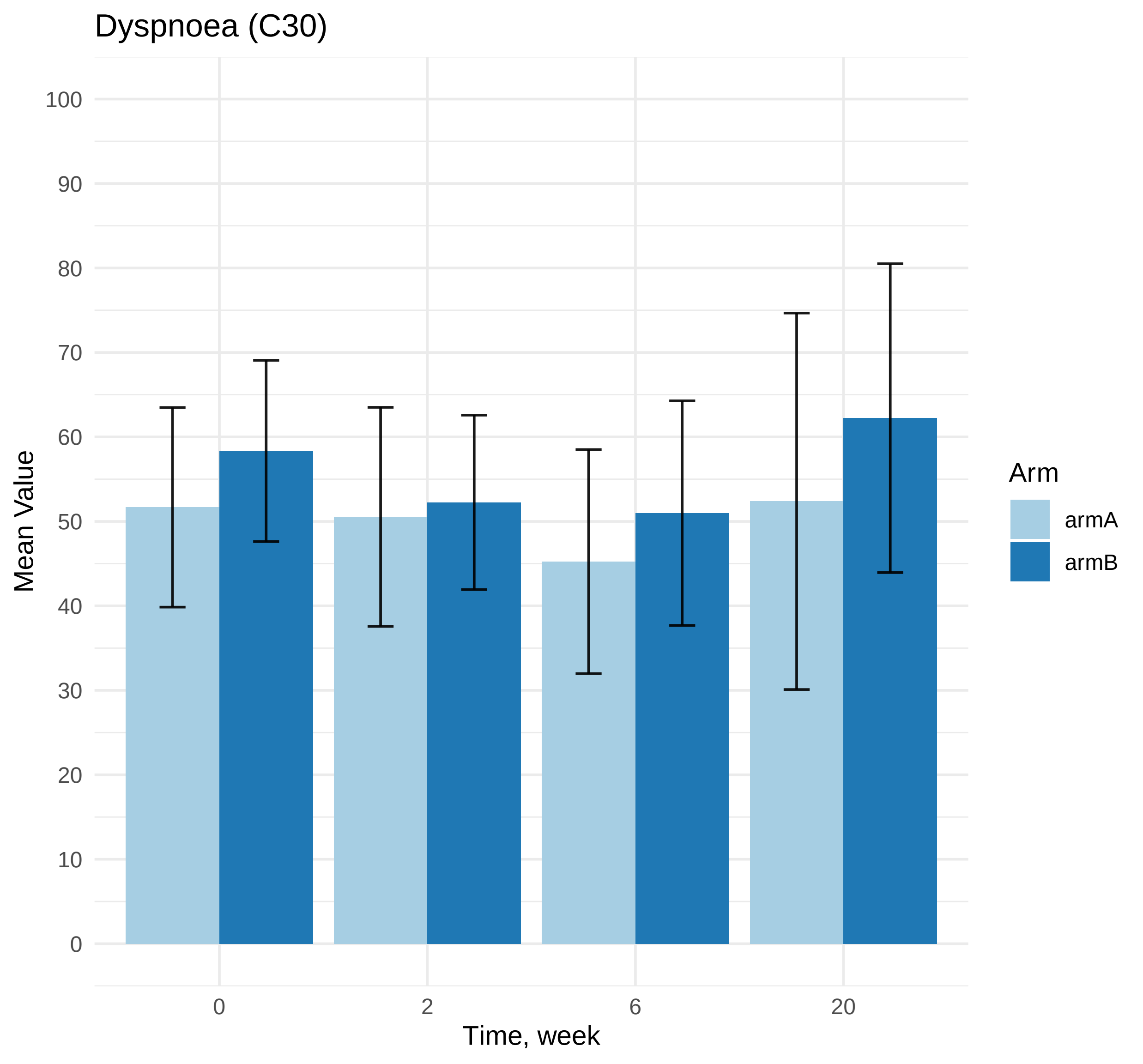
Dyspnoea (C30)
Mean Value
Time, week
Arm
armA
armB
0
10
20
30
40
50
60
70
80
90
100
0
2
6
20

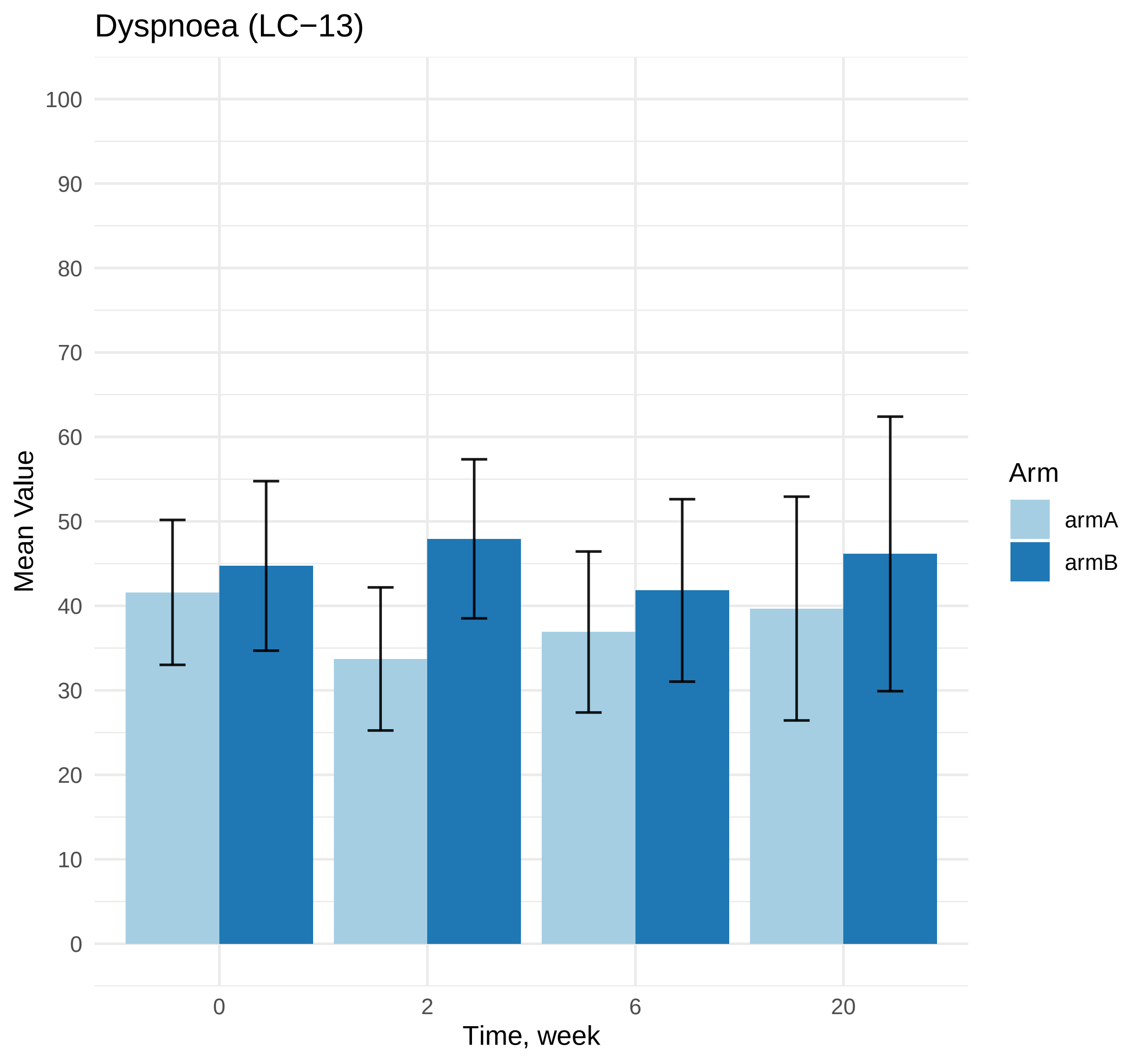

Dyspnoea (LC−13)
Mean Value
100
90
80
70
60
50
40
30
20
10
0
0
2
6
20
Time, week
Arm
armA
armB

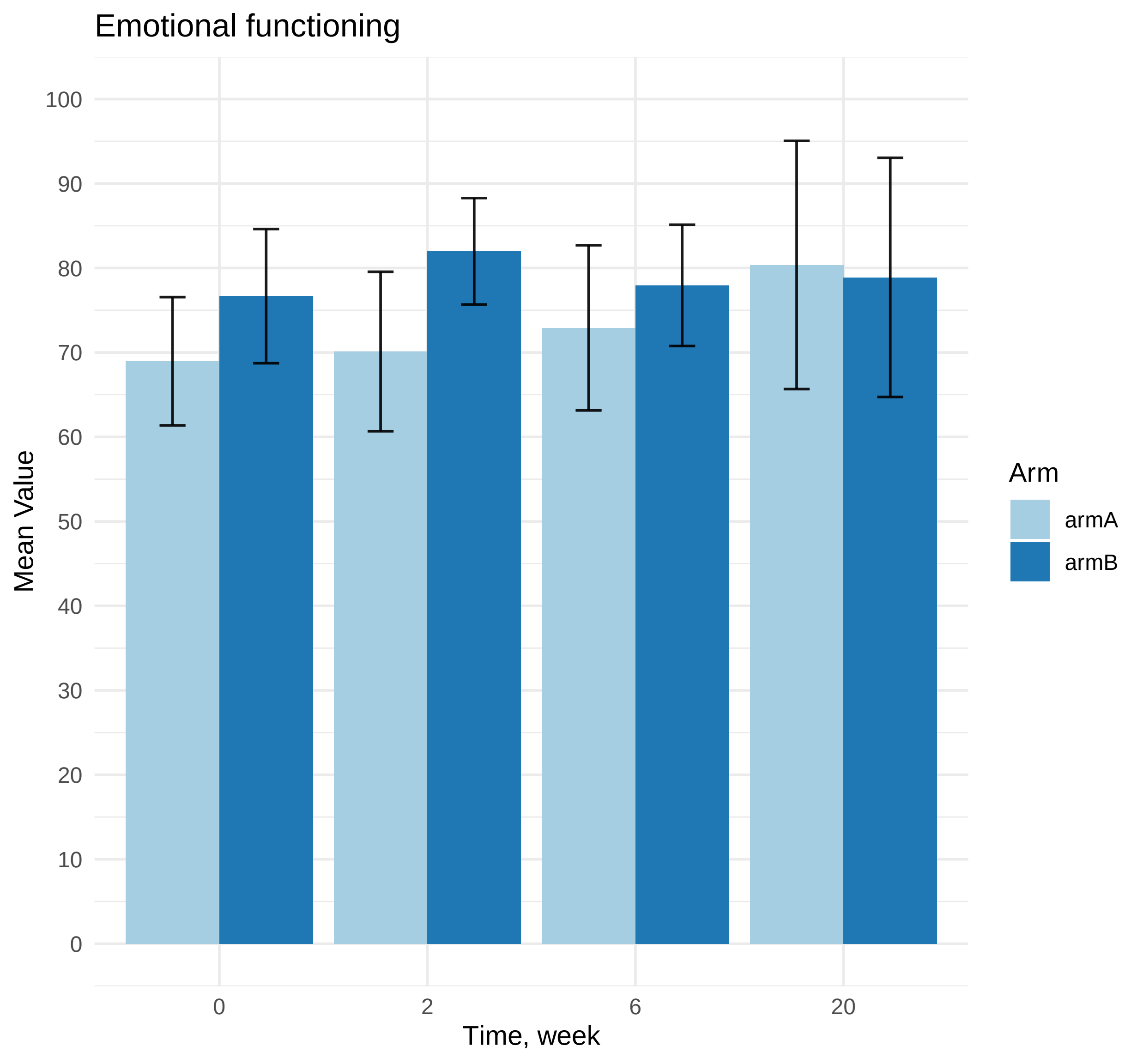
Emotional functioning
Mean Value
100
90
80
70
60
50
40
30
20
10
0
0
2
6
20
Time, week
Arm
armA
armB

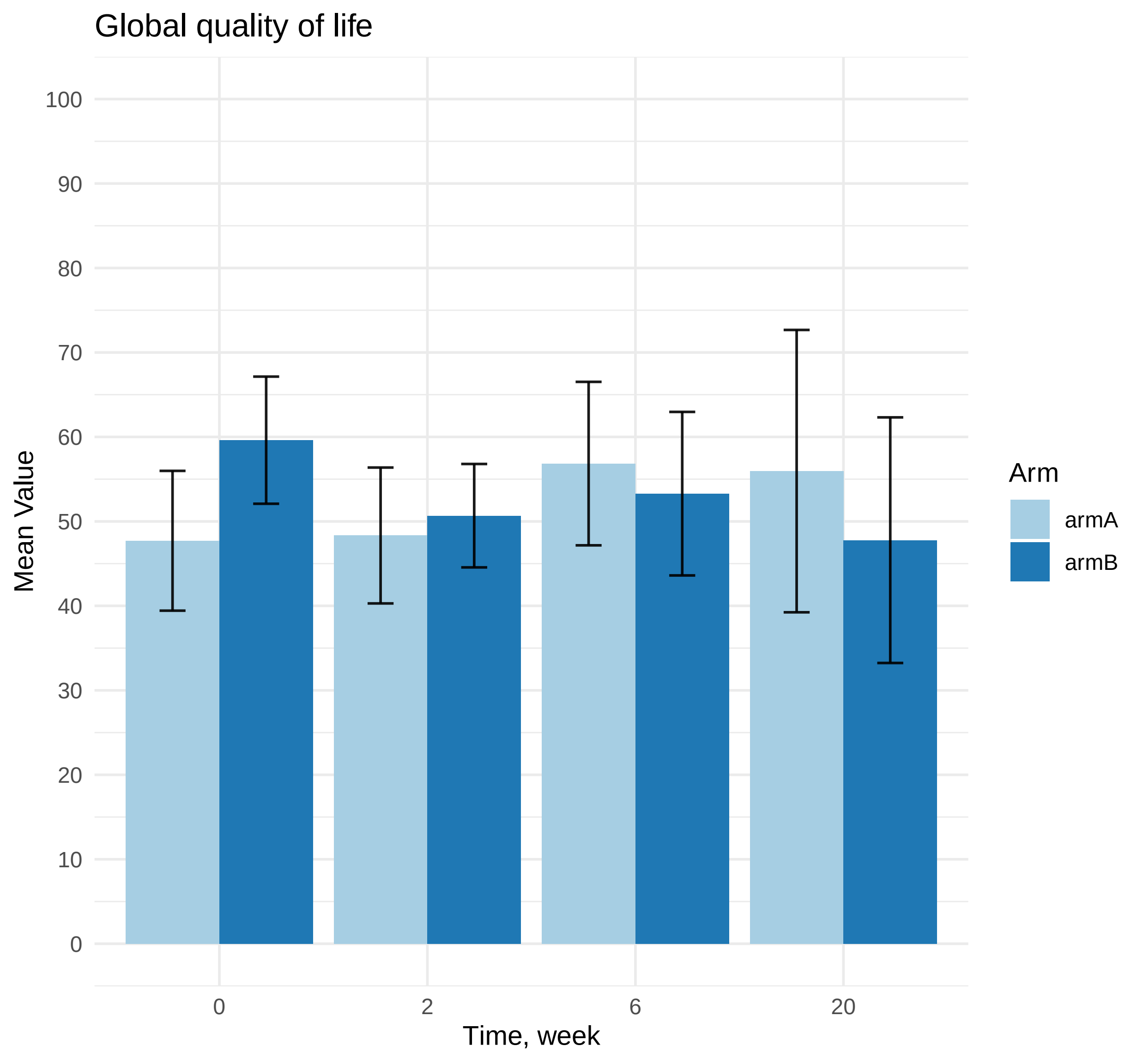
Global quality of life
Mean Value
Time, week
0
2
6
20
0
10
20
30
40
50
60
70
80
90
100
Arm
armA
armB

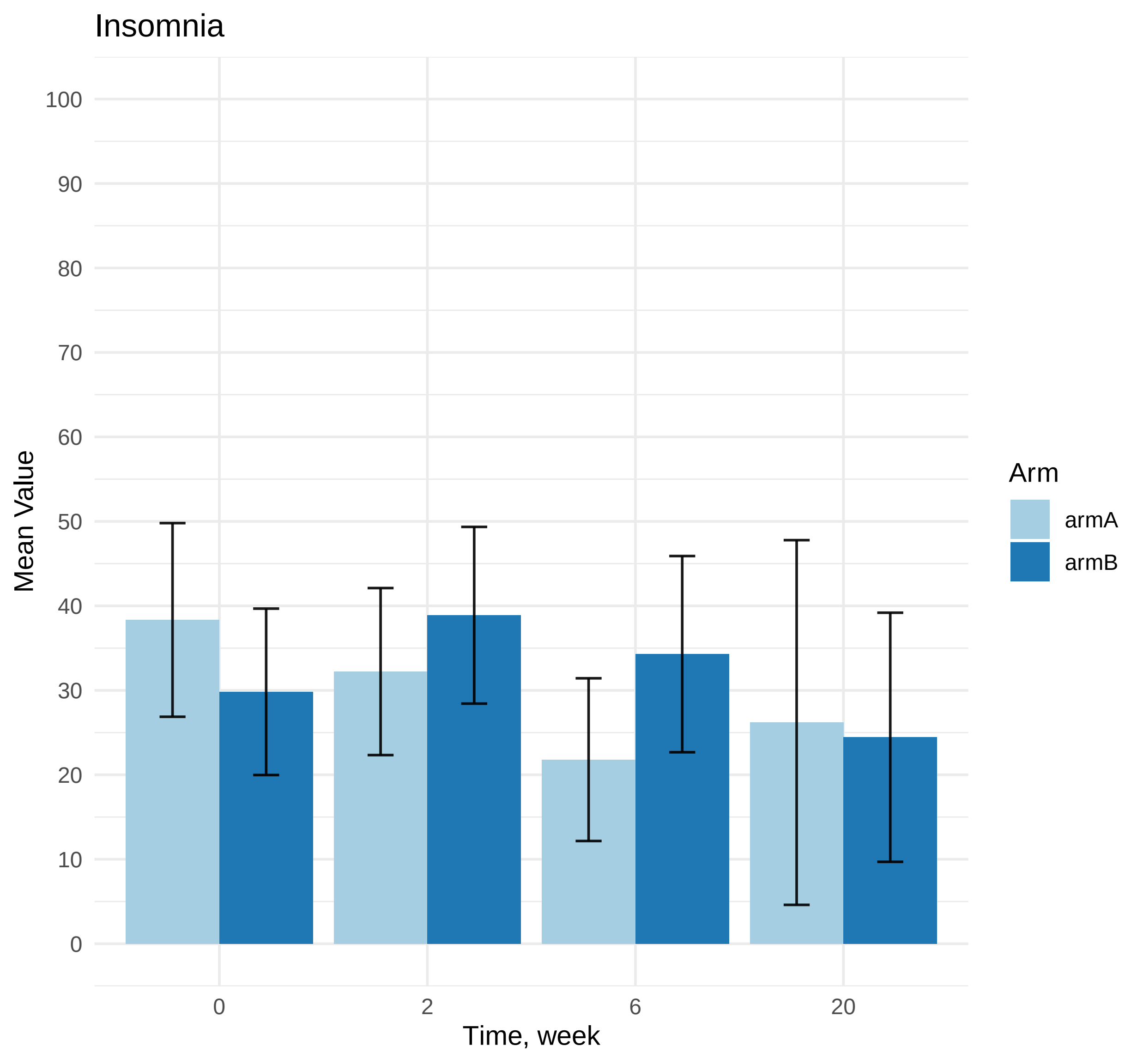

Insomnia
Mean Value
0
10
20
30
40
50
60
70
80
90
100
0
2
6
20
Time, week
Arm
armA
armB

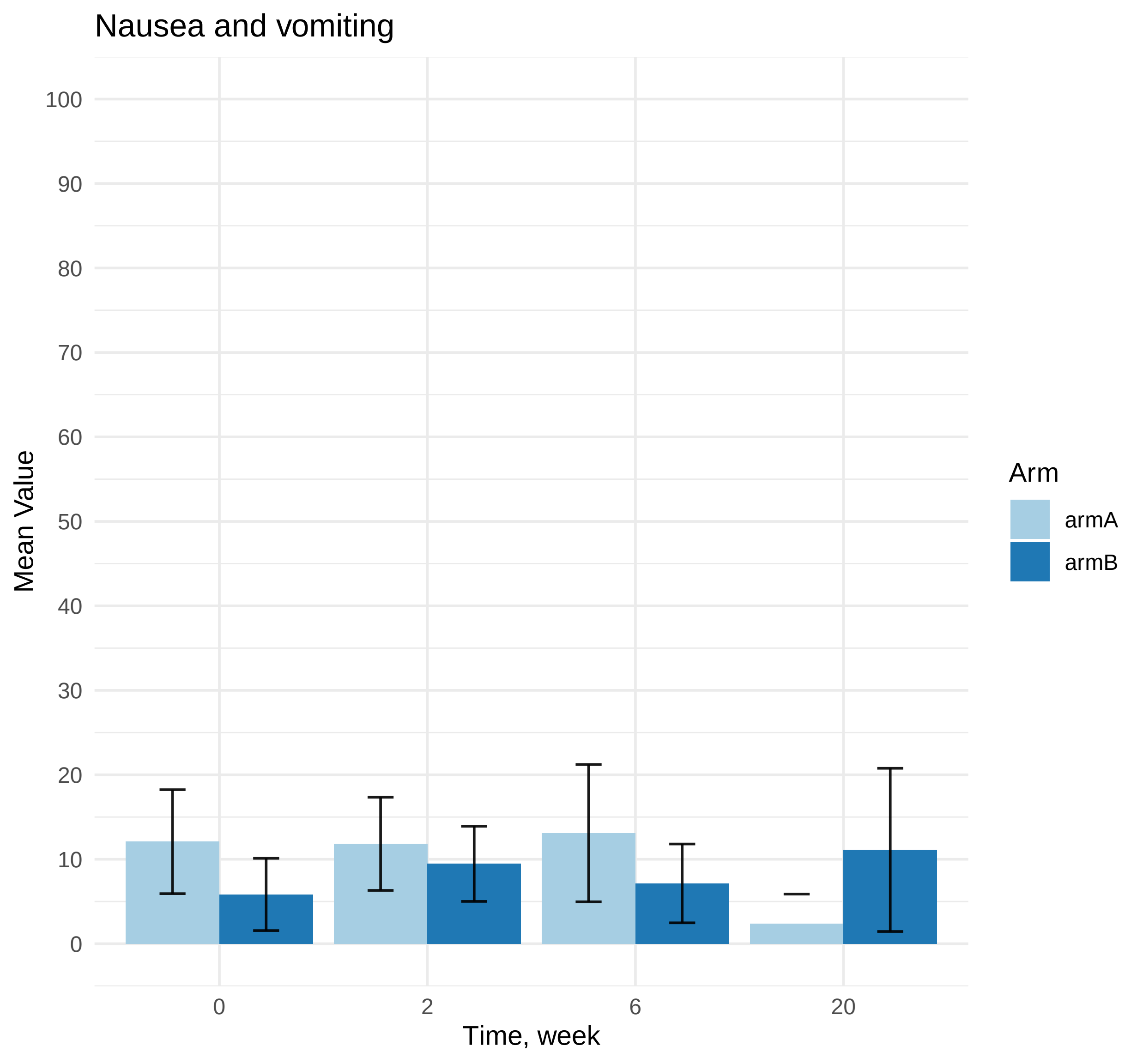

Nausea and vomiting
Mean Value
100
90
80
70
60
50
40
30
20
10
0
0
2
6
20
Time, week
Arm
armA
armB

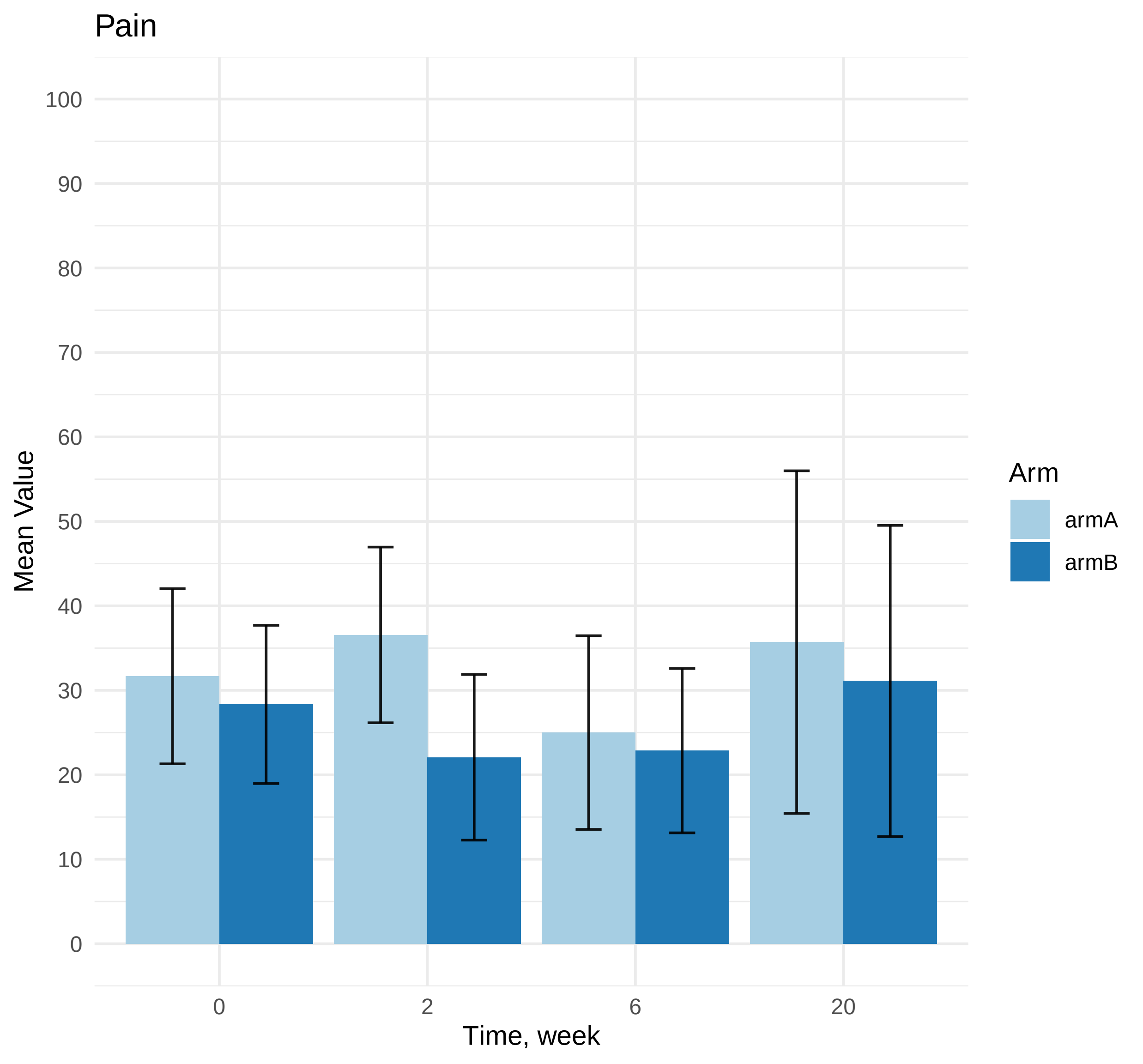
Pain
Mean Value
100
90
80
70
60
50
40
30
20
10
0
0
2
6
20
Time, week
Arm
armA
armB

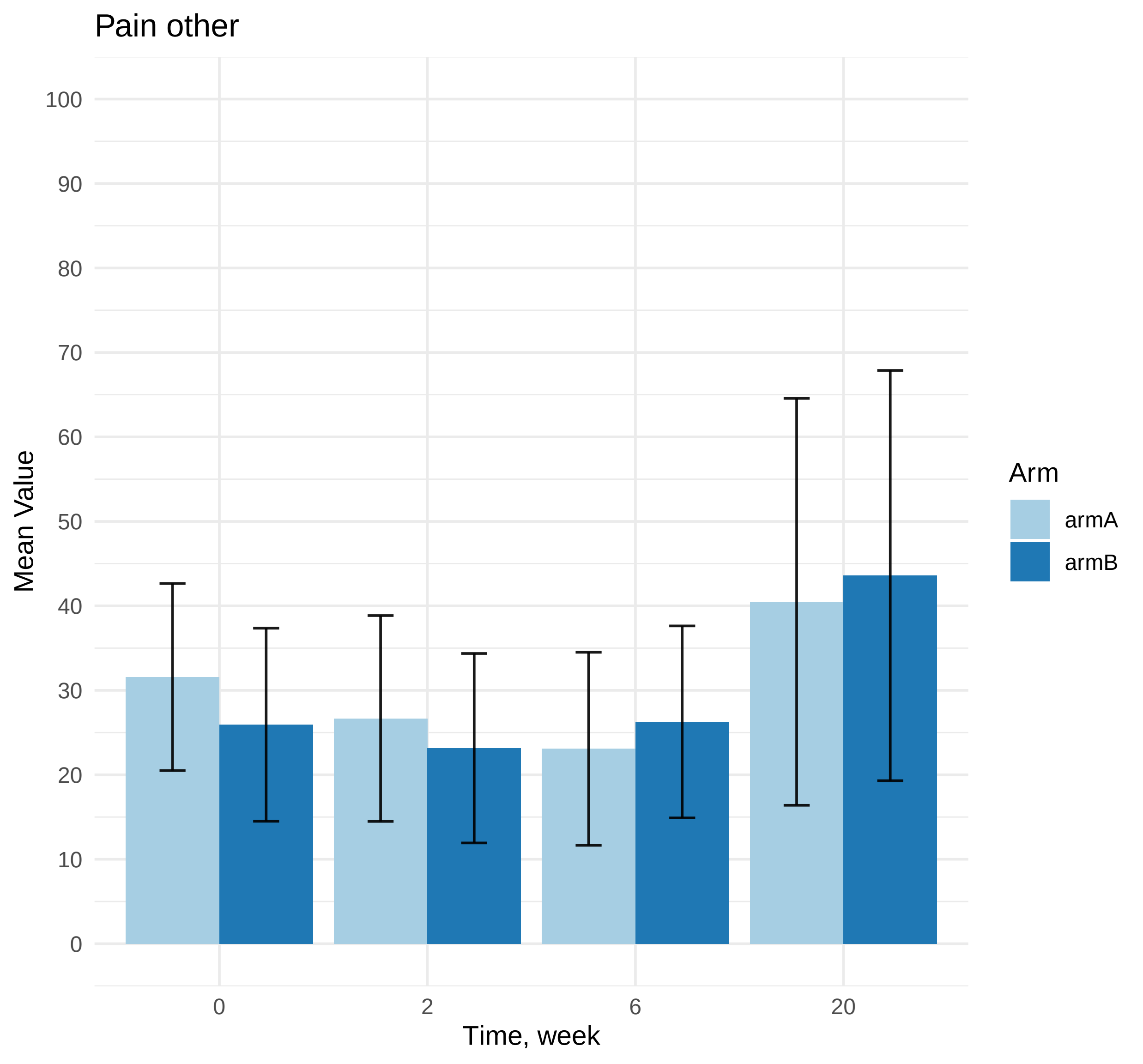

Pain other
Mean Value
100
90
80
70
60
50
40
30
20
10
0
0
2
6
20
Time, week
Arm
armA
armB

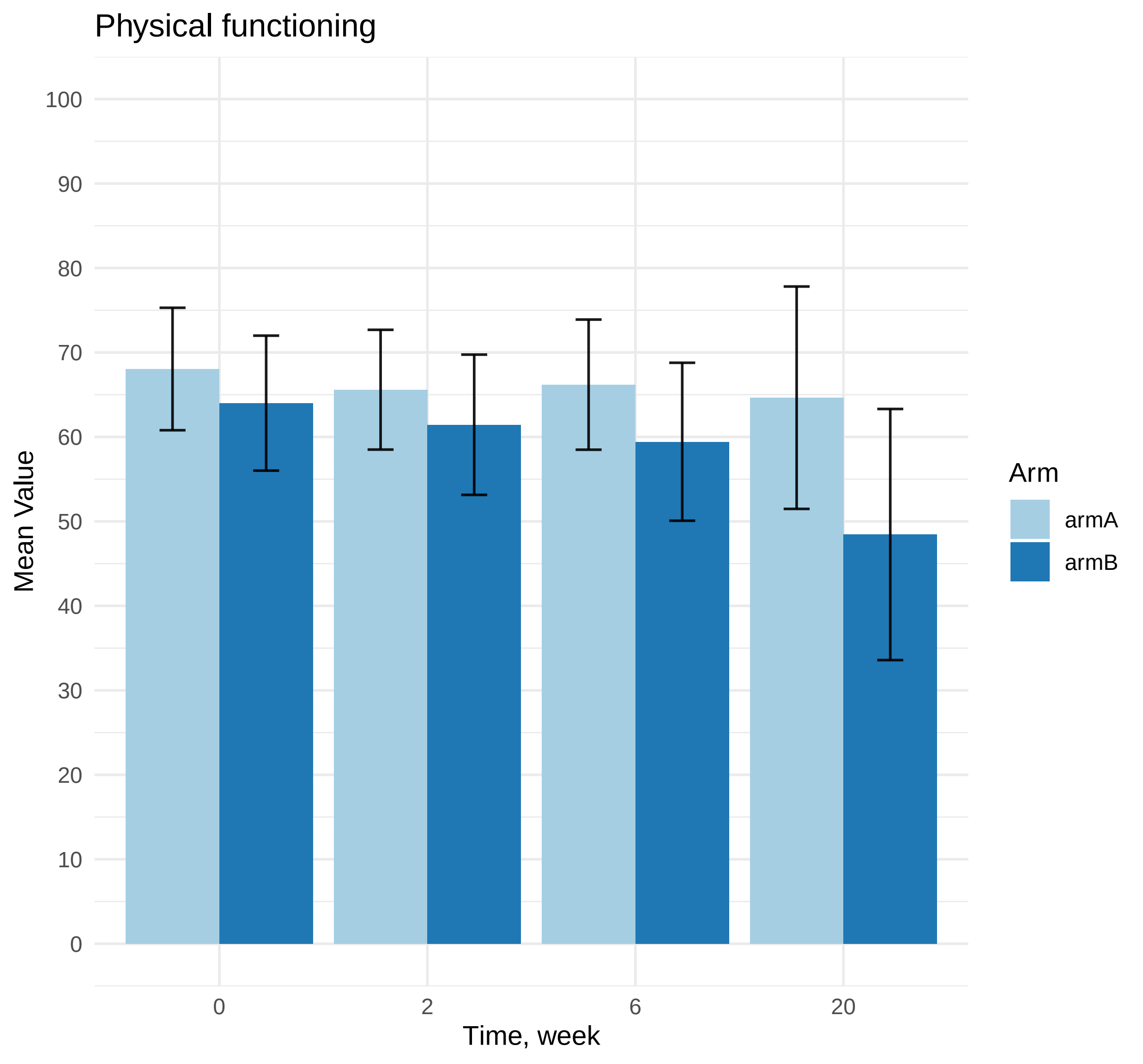

Physical functioning
Mean Value
100
90
80
70
60
50
40
30
20
10
0
0
2
6
20
Time, week
Arm
armA
armB

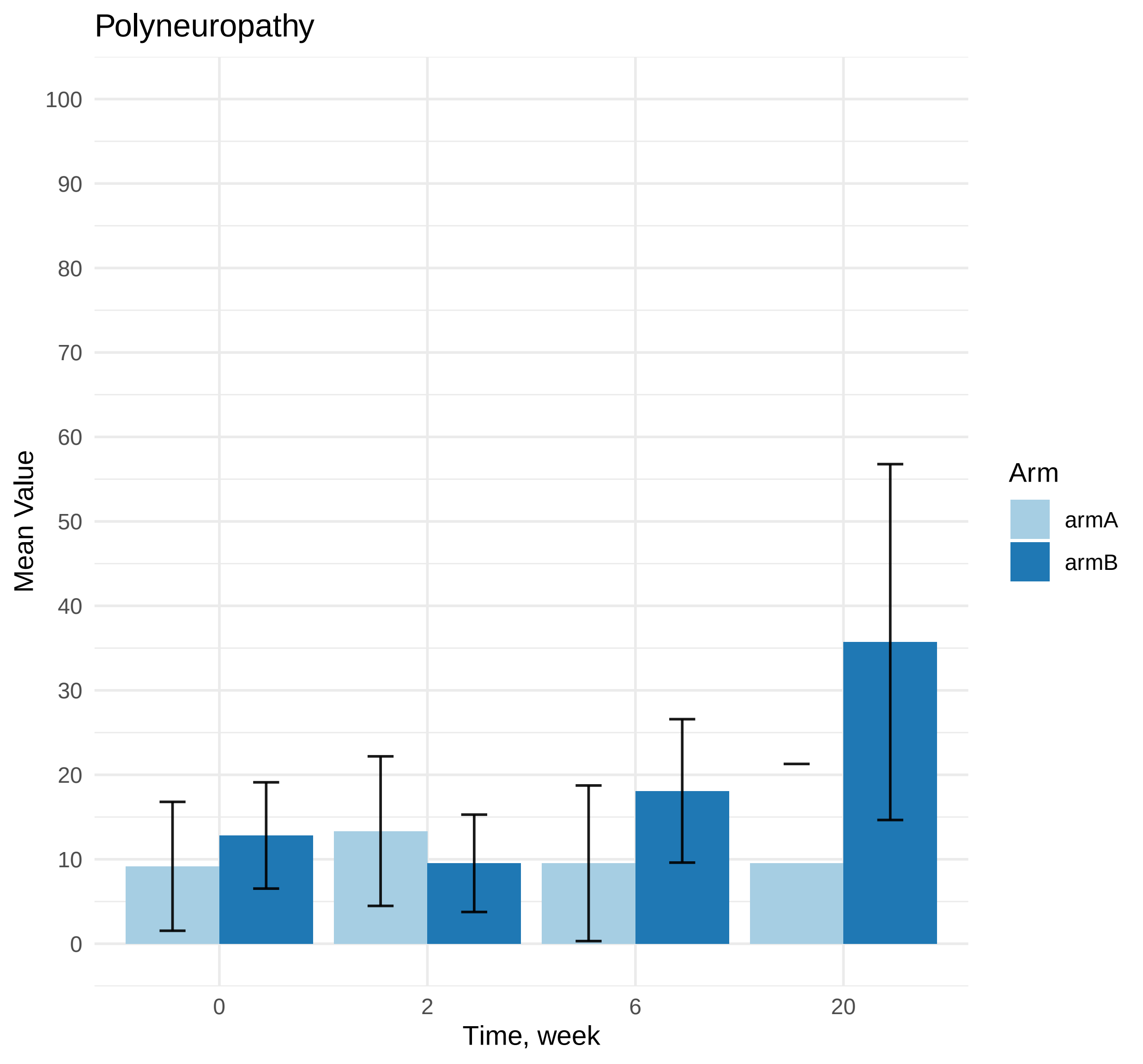

Polyneuropathy
Mean Value
100
90
80
70
60
50
40
30
20
10
0
0
2
6
20
Time, week
Arm
armA
armB

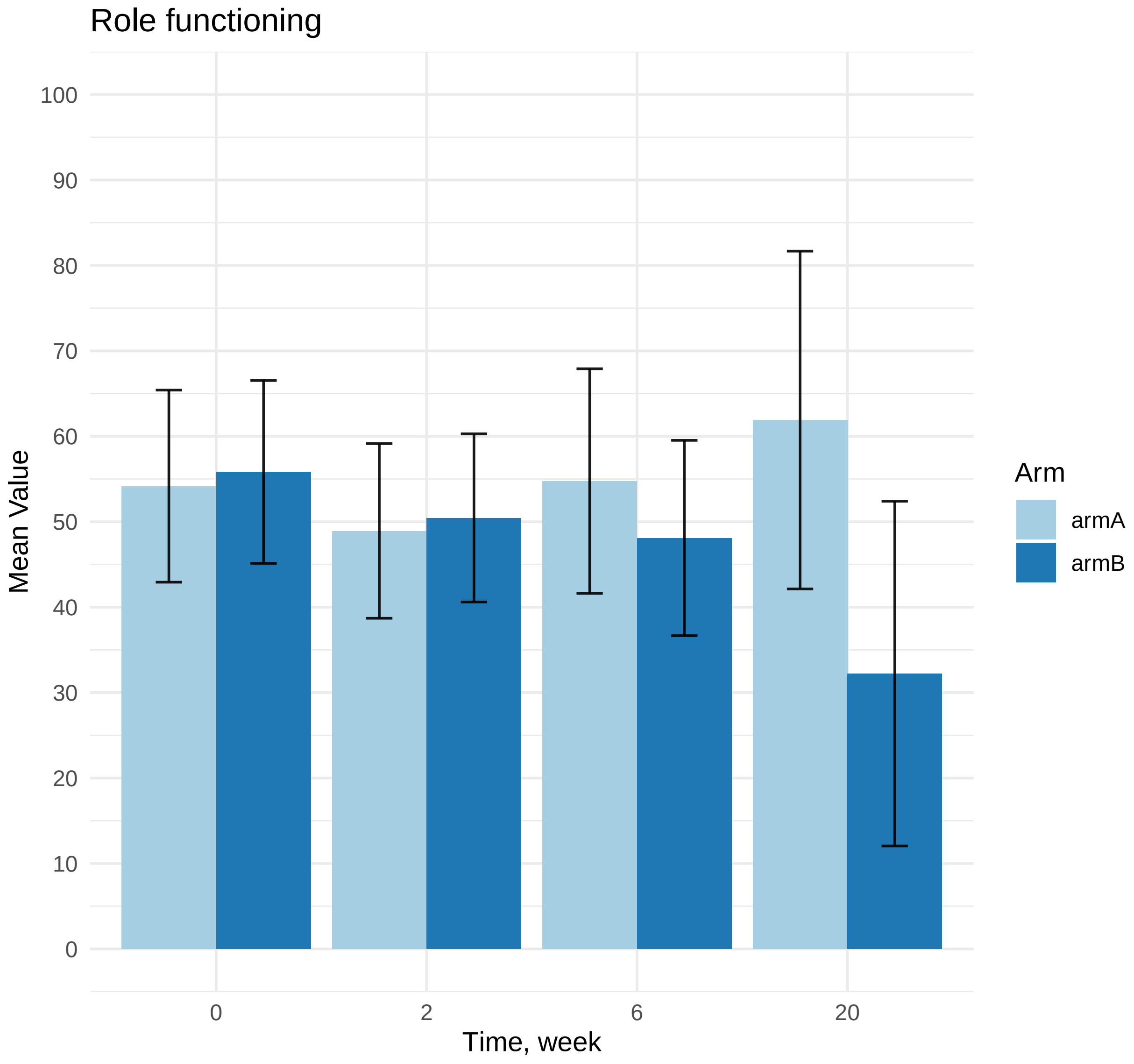

Role functioning
Mean Value
100
90
80
70
60
50
40
30
20
10
0
0
2
6
20
Time, week
Arm
armA
armB

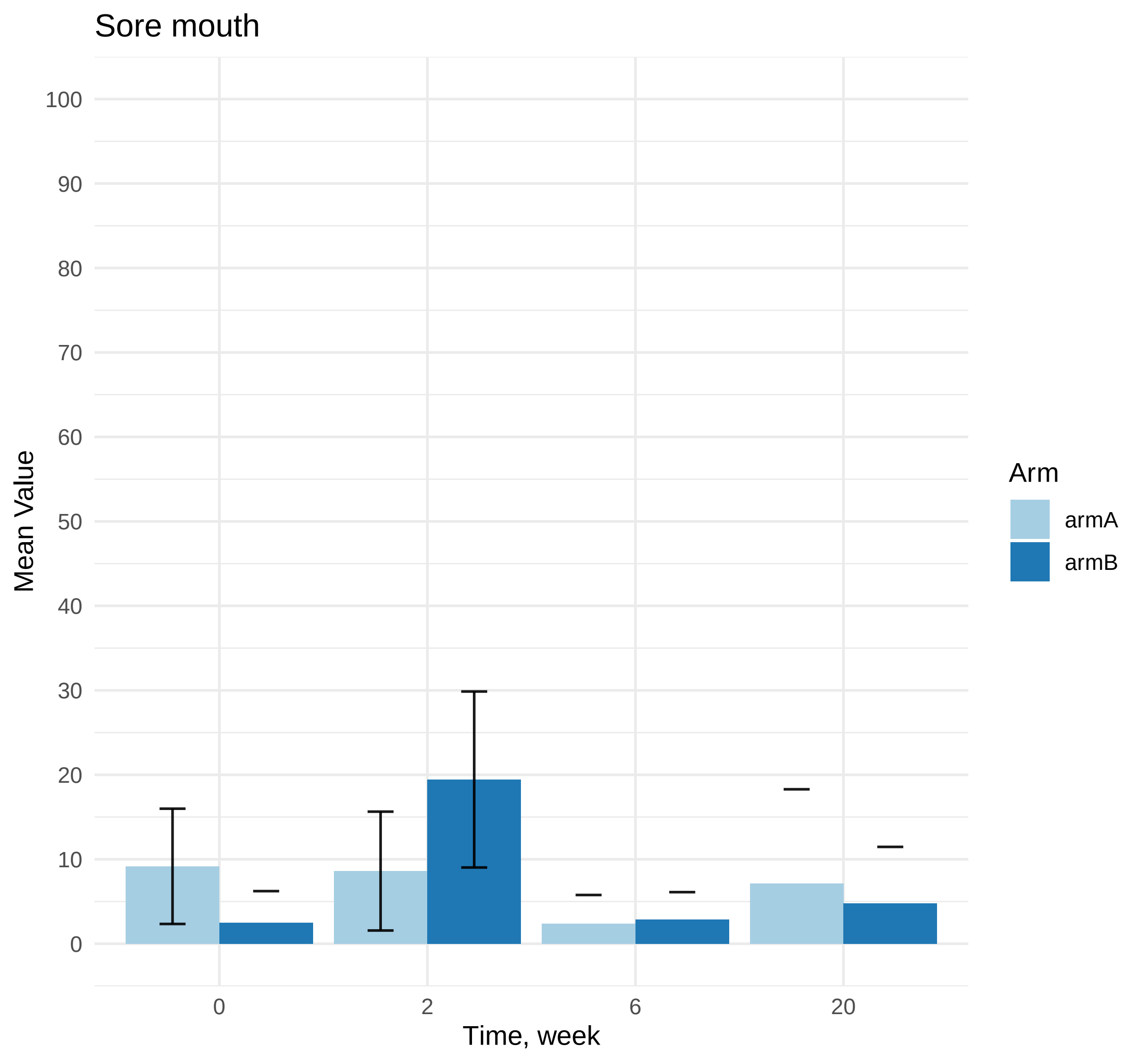
Sore mouth
Mean Value
0
10
20
30
40
50
60
70
80
90
100
Time, week
0
2
6
20
Arm
armA
armB

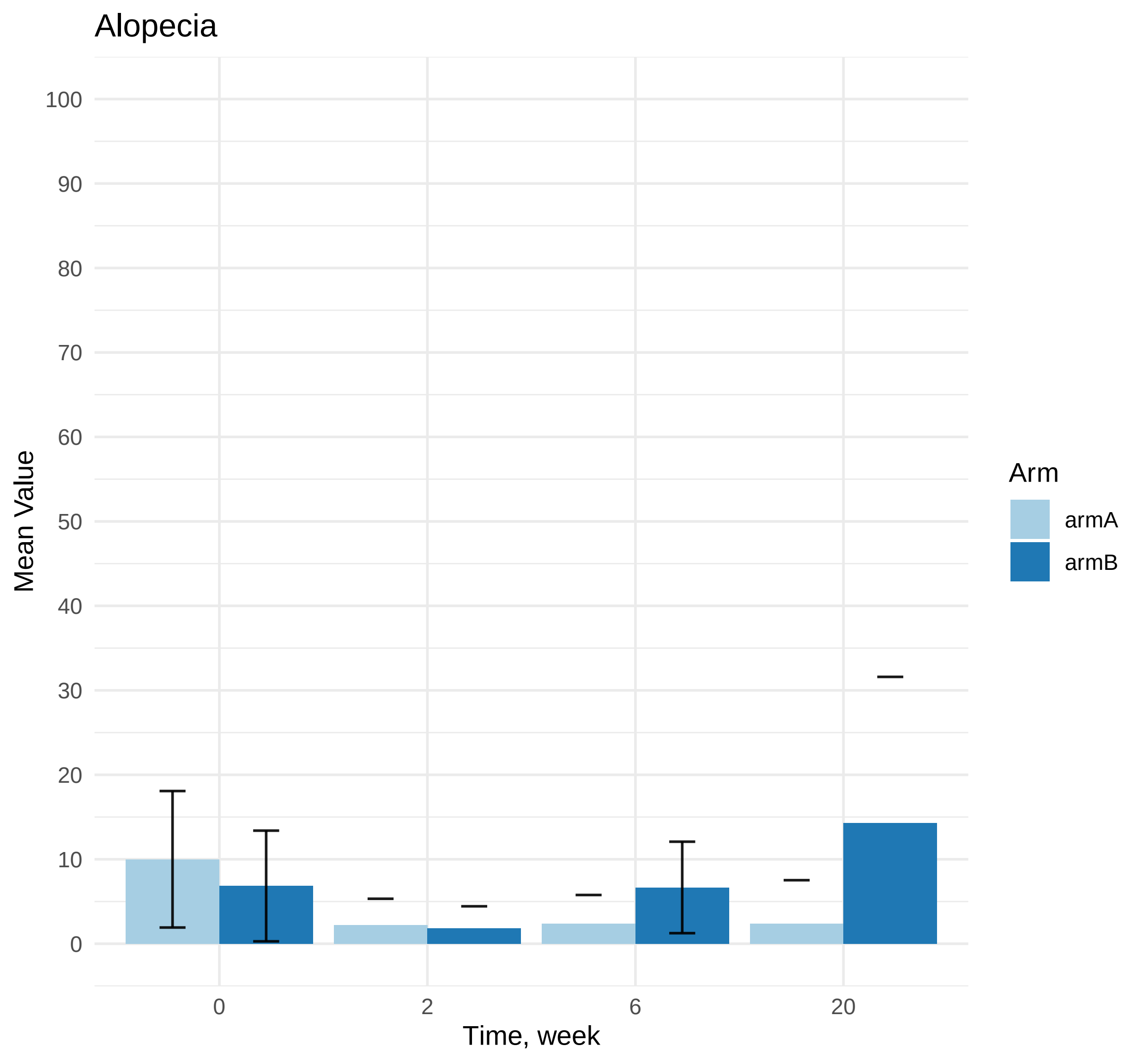
Alopecia
Mean Value
100
90
80
70
60
50
40
30
20
10
0
0
2
6
20
Time, week
Arm
armA
armB

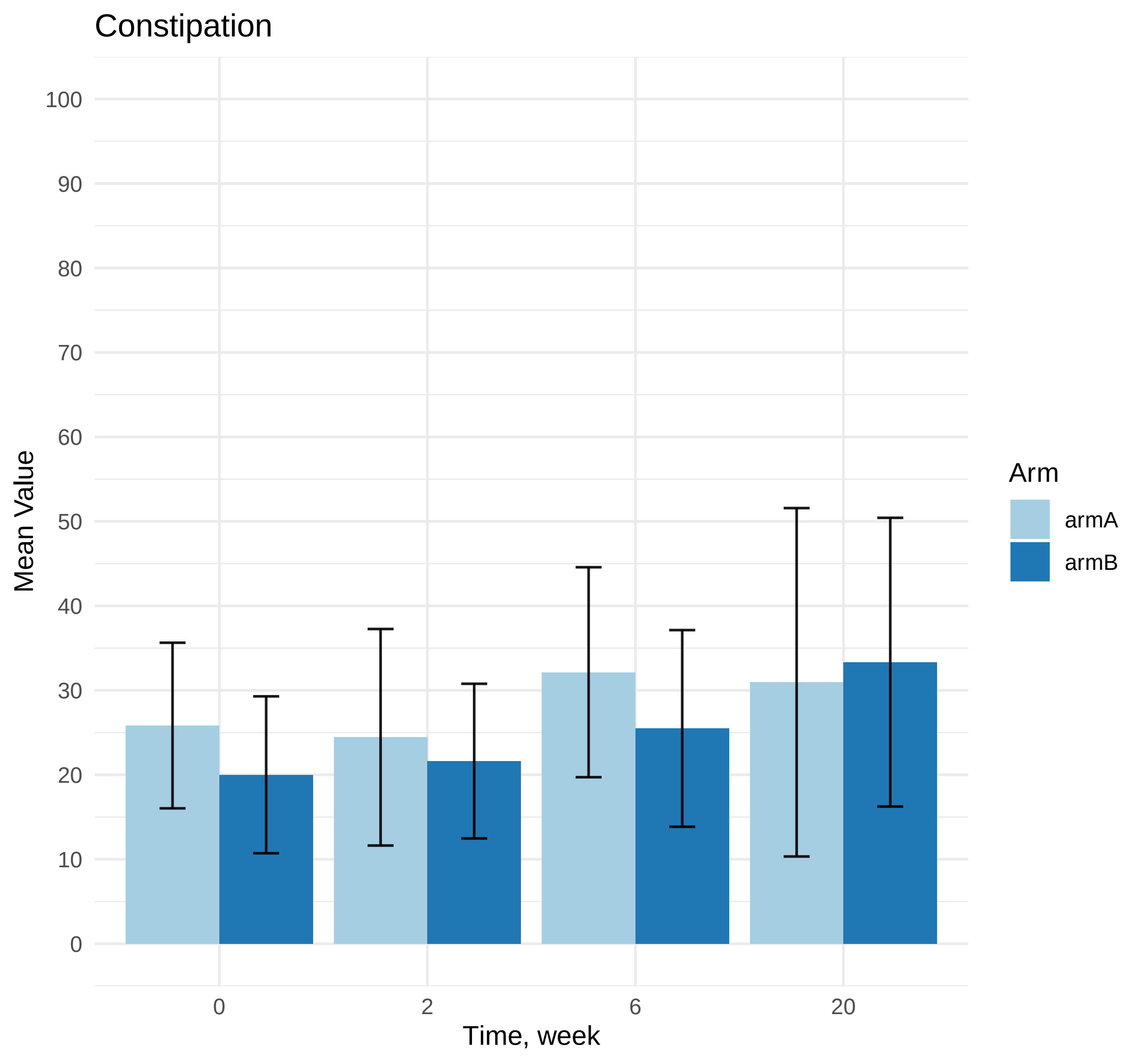

Constipation
Mean Value
100
90
80
70
60
50
40
30
20
10
0
0
2
6
20
Time, week
Arm
armA
armB

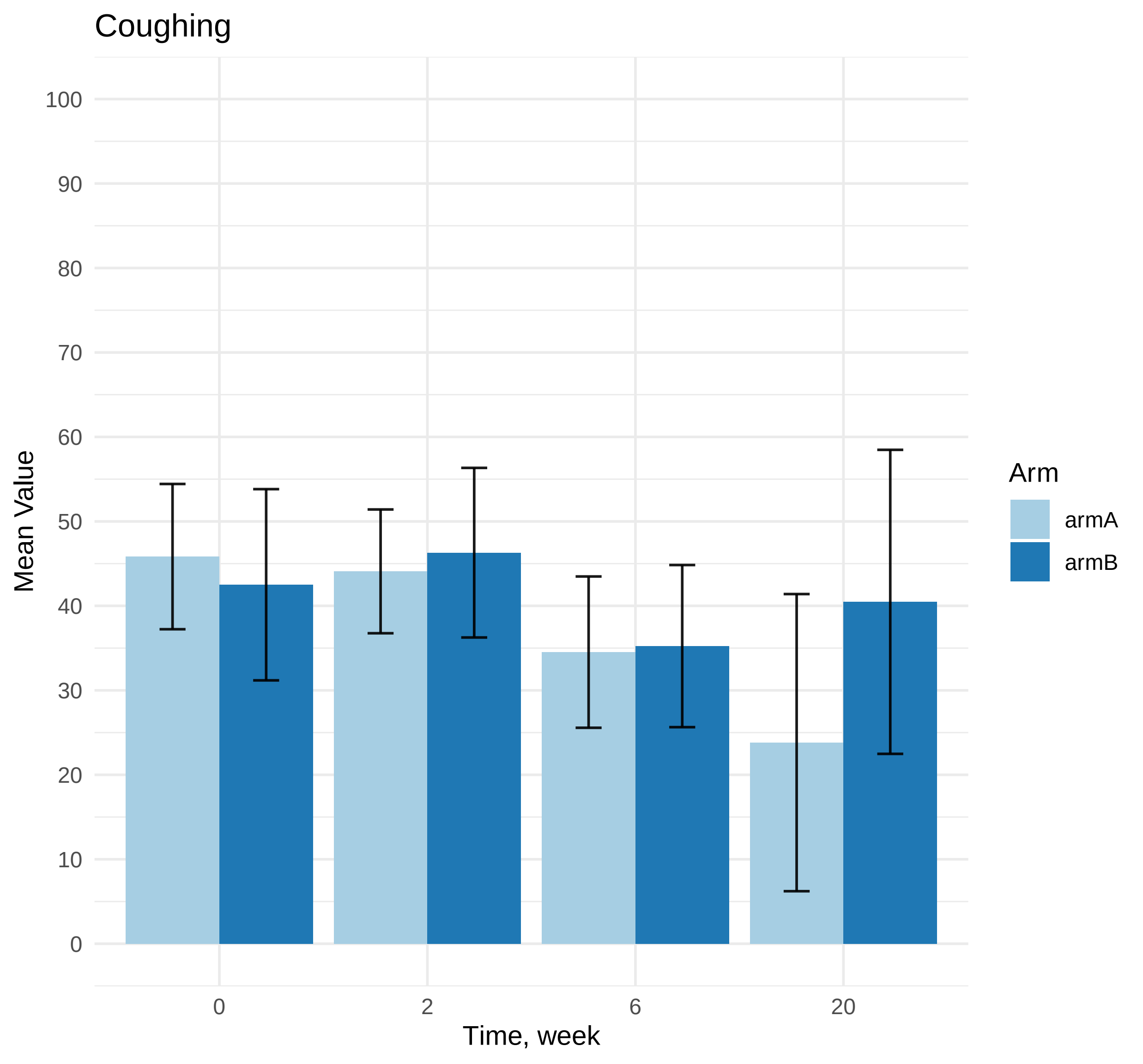
Coughing
Mean Value
100
90
80
70
60
50
40
30
20
10
0
0
2
6
20
Time, week
Arm
armA
armB

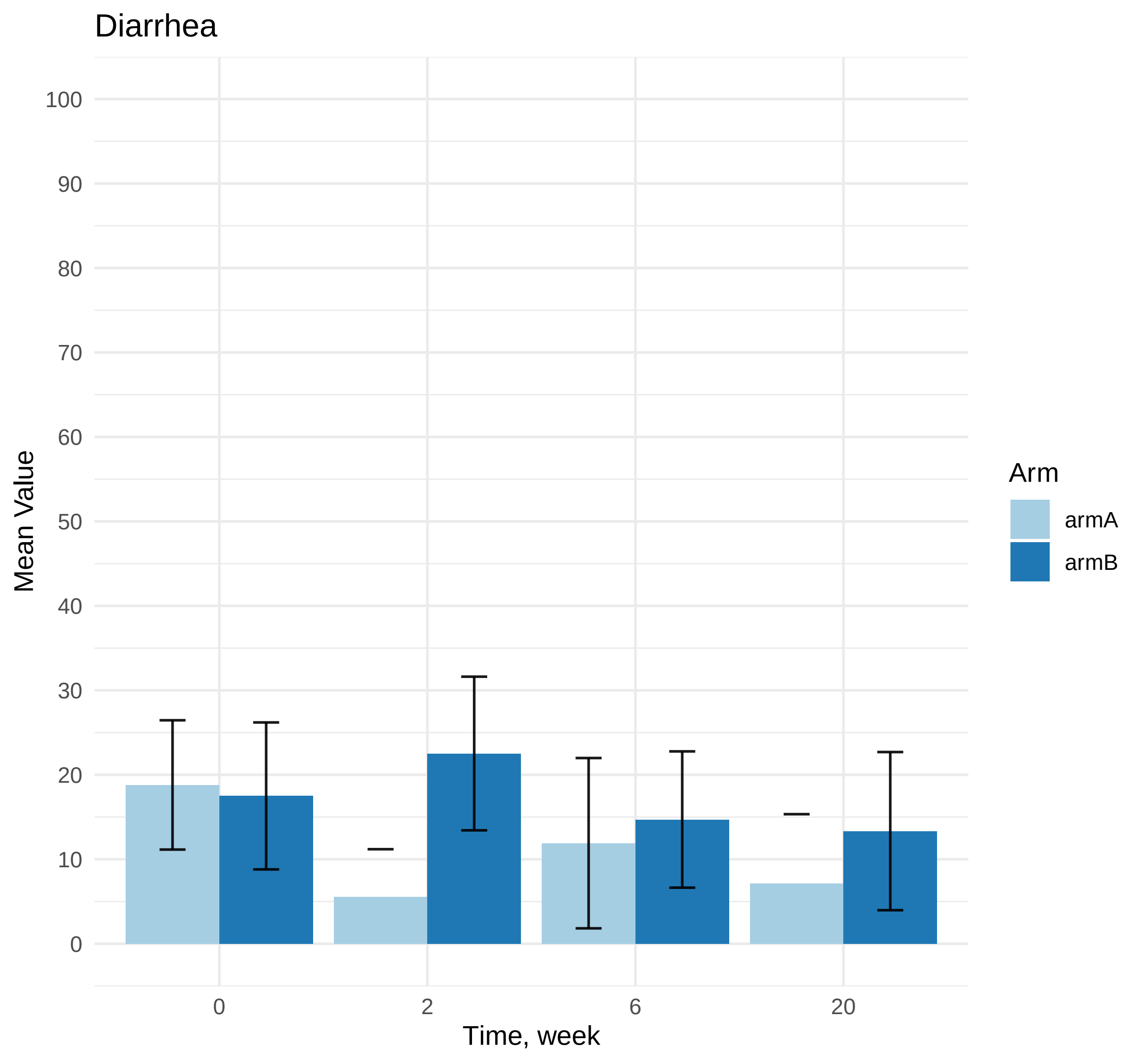

Diarrhea
Mean Value
100
90
80
70
60
50
40
30
20
10
0
0
2
6
20
Time, week
Arm
armA
armB
